# Influence of Simple and Double-Weave Structures on the Adhesive Properties of 3D Printed Fabrics

**DOI:** 10.3390/polym14040755

**Published:** 2022-02-15

**Authors:** Marjeta Čuk, Matejka Bizjak, Tanja Nuša Kočevar

**Affiliations:** Department of Textiles, Graphic Arts and Design, Faculty of Natural Sciences and Engineering, University of Ljubljana, Snežniška Ulica 5, 1000 Ljubljana, Slovenia; matejka.bizjak@ntf.uni-lj.si (M.B.); tanja.kocevar@ntf.uni-lj.si (T.N.K.)

**Keywords:** 3D printing, adhesion, simple weave, double weave, morphology, z-distance

## Abstract

The double-weave structure of a fabric allows for the use of different materials and weave structures for the upper and lower layer, which can be advantageous in the functionalization of 3D printed textiles. Therefore, the aim of this research was to investigate the influence of simple and double-weave structures on the adhesion of 3D printed fabrics. From this perspective, we investigated the influence of different twill derivates and weft densities on the adhesion force. We produced fabrics specifically for this study and printed them with a polylactic acid filament using Fused Deposition Modeling technology. The T-peel test was performed to measure the adhesion, and the results were statistically analyzed. A morphological study of the surfaces and cross-sections of the 3D printed fabrics helped us interpret the results. We found that adhesion was higher for double fabrics when printed with a smaller z-distance, where the molten polymer reached the lower layer of the fabric and adhered to it. The opposite was confirmed when printing with a larger z-distance, where adhesion was higher for simple fabrics. Both weave and density had a significant effect on adhesion in all cases. Surprisingly, different twill derivatives generally had a greater influence on adhesion than density.

## 1. Introduction

Recently, we have witnessed an expansion in the development of various technologies that open up new opportunities for interdisciplinary connections. Among them, there are also 3D printing technologies, which were initially used in the fashion industry, but are now spreading to other areas of textile and apparel design and production [1]. In the last decade, as 3D printing has become established in the textile and fashion industry, the number of high-quality research papers has also increased rapidly.

Three-dimensional printing is an additive manufacturing (AM) technology that produces three-dimensional objects by depositing layers of material. Depending on the 3D printing technology and the material used, these layers are bonded together in different ways [2,3]. Among its many applications in the field of textile production, such as printing flexible structures to replicate some textile properties with rigid materials [4] and printing textile-like structures with flexible materials, 3D printing directly on textiles is currently the most commonly used process [5]. For this process, the polymer-based AM technique Fused Deposition Modeling (FDM) is most commonly used.

In FDM technology, the 3D printer extrudes thermoplastic filaments based on the designed 3D model, precisely controlling the temperature and speed of the polymer flow [6]. The most common polymer used for 3D printing with FDM technology is polylactic acid (PLA). Other polymers used in 3D printing include acrylonitrile butadiene styrene (ABS), polycarbonate (PC), and blends of PC and ABS [7]. PLA is a synthetic aliphatic polyester derived from agricultural raw materials and is classified as a biopolymer due to its renewability and degradability in nature. PLA can be extruded at a relatively low temperature (about 210 °C), which means that the filaments can be more easily extruded through the FDM nozzle compared with other building materials [8].

Due to the wide range of functions that can be achieved with 3D printed structures on flexible and soft fabrics, a variety of uses for printed multi-material objects on the textile substrate are possible. Three-dimensional printing on textile surfaces makes it possible to customize the product. In the field of clothing production, this means customizing a piece of clothing or the entire garment, resulting in an increase in the functionality of the garment. This type of functionalization is important in the field of sports and personal protective equipment, as 3D printing of protective and support elements enables the production of functional clothing that perfectly fits the body [9,10].

The main drawback and at the same time the main challenge is to achieve sufficient adhesion of the 3D printed material to the textile surface and to maintain the flexibility of the fabric. Therefore, the main focus of the numerous research groups has been the study of the parameters on which the adhesion depends.

Adhesion of the polymer to the substrate occurs through three main adhesion mechanisms, namely mechanical coupling, molecular bonding, and thermodynamic adhesion [11]. In studies, it has been found that when PLA or ABS polymers are printed on various textile substrates such as PES, nylon, wool, or wool/PES, physical interlocking bonds are formed without any chemical bonding between the polymer and the substrate material [12].

Research on the adhesion of 3D printed objects to the textile substrate has been conducted in the following main areas: influence of the 3D printing process, influence of textile substrate parameters, such as fabric construction parameters, and influence of pre- and post-treatment of 3D printed fabrics. The research is carried out on woven and knitted fabrics. When printing on textiles with large open areas, such as net fabrics, adhesion is not a problem, especially when the polymer penetrates through the open pores and embeds the yarns into the molten material, building form-locking connections [13,14]. Porous textile surfaces, which are more common in knitted than woven fabrics, are therefore more conducive to a high degree of adhesion of 3D printed objects to textile surfaces [15].

One of the most important printing parameters affecting adhesion to textile substrates is the distance between the nozzle and the printing bed, called the z-distance. If we decrease the distance, the adhesion force increases until the minimum distance is reached, and the filament clogs the nozzle. With a smaller distance, the nozzle pushes the polymer into the pores of the fabric with higher force [16]. It was also found that the effect of z-distance on adhesion depends on the material used [12]. Moreover, the adhesion force depends on different orientations of the filling and also on the orientation of the first printed layer, with adhesion forces increasing from 0° to 90° and decreasing when the angle of infill is larger. An orientation of 0° means that the first layer infill is printed parallel to the long side of the sample, and 90° means that the first layer infill is printed parallel to the short side of the sample [17]. Three-dimensional printing process parameters that affect adhesion include printing bed temperature and extruder temperature. When you increase both temperatures, the viscosity of the molten filament decreases during printing, causing the material to penetrate deeper into the fabric [16,18].

When studying the adhesion force of 3D printed objects on the textile substrate, the influence of the fabric construction parameters also proved to be very important. In terms of weave pattern and weft density, Malengier et al. found that plain weave exhibited the lowest adhesion compared with twill and satin weave, and that a higher weft density influenced the improvement of adhesion properties for twill and satin weave, with the substrate with twill weave confirmed to be optimal [19]. Better adhesion can also be achieved when the textile surface is roughened or hairy, and better adhesion forces can also be obtained with thicker fabrics. These good adhesion results can be attributed to the intended interlocking bonds of the printed polymer with the fibers on the top of the textile as well as inside the textile structure, which should provide sufficient open areas for penetration of the molten polymer [9].

The pre- and after-treatment of textile substrates for 3D printed textiles have also been confirmed to have an effect on adhesion strength. Some pre-treatment processes, such as chemical pre-treatments [17] and polymer coatings on textiles [20], improved the adhesion between the printed polymer and the textile substrate, but, in contrast, washing prior to the 3D printing process can also reduce adhesion in some cases [21]. As an after-treatment, thermal treatment of 3D printed textiles by ironing was investigated and the results showed that it has a significant effect on adhesion [22].

From the previously published research results and our preliminary investigations, it is clear that the surface of the fabric greatly affects the adhesive properties, which are mainly related to the weave type and thread density. Therefore, the aim of this research was to investigate in more detail the influence of these two parameters and the surface morphology of the fabric on the adhesion properties, as well as the influence of some printing parameters.

The experimental part of this study was divided into two parts. First, we investigated the influence of different fabric constructions on the adhesion of 3D printed objects. For this purpose, we produced woven fabrics under controlled conditions, with planned weave structures: simple and double fabrics with different weave patterns and weft densities. Our main interest was to investigate the difference between simple and double weaves and their influence on the adhesion strength of 3D printed objects. There are many studies in the literature explaining the influence of simple weave structures on the adhesion of a 3D printed polymer to the substrate, but, to our knowledge, no study has been published on double-weave fabrics where the threads are arranged in a completely different way. Second, we studied the morphology of 3D fabrics printed with a PLA polymer with different z-distances. We observed the penetration of the molten PLA polymer as a function of the different weaves and weft densities.

The results of the study show that the adhesion was higher for double fabrics and that the weave and density had a large effect on adhesion.

## 2. Materials and Methods

### 2.1. Materials

Twelve woven fabrics were designed and produced for the study. Three simple fabrics were designed in different twill weaves and with two different weft densities, and three double fabrics were designed in different double twill weaves and with two different weft densities, all using the CAD weaving program ArahWeave (Arahne d.o.o, Ljubljana, Slovenia).

Simple weave fabrics consist of a single set of warp threads and a single set of weft threads interwoven in a weave pattern, creating a single-ply fabric. Double woven fabrics are made of two sets of warp threads and two sets of weft threads, in our case, creating a double-ply fabric.

For all woven samples, black and white 100% cotton yarns (8 × 2 tex) were used for the warp and blue 100% polyester yarn (33 tex) was used for the weft. The preset warp density on the loom was 40 threads/cm, and the preset weft density on the loom was 15 and 20 threads/cm for simple weaves, and 30 and 40 threads/cm for double weaves. All samples were woven on a Minifaber sample loom (Minifaber, Seriate, BG, Italy) with an electronic jacquard TIS (TIS Electronics, Beligneux, France) with the same production settings. The warp threads were not sized before weaving and the fabrics were not pre-treated before 3D printing. Table 1 lists all fabric samples and their designations.

The designations were created according to the weave structure and weft density of the fabrics (Table 1). Fabrics woven in double weave are designated D, twill 1/3 in the z direction is designated T13Z, twill 2/2 in the z direction is designated T22Z, and broken twill 1/3 is designated T13B. A preset weft density of 15 threads/cm is marked 15, a weft density of 20 threads/cm is marked 20, 30 threads/cm is marked 30, and 40 threads/cm is marked 40. The fabrics were printed with different z-distances. When the z-distance was constant, samples were labeled z1 and when they were printed with a constant z-distance offset, they were labeled z2.

The names of the samples are used in some cases to denote the fabrics, e.g., T13Z, and in cases where density is important, the names of the samples have numbers, e.g., T13Z 15. In cases where z-distance (3D printed samples) is discussed, the names of the samples have an appendix, e.g., T13Z 15-z1.

After weaving, the physical properties of the fabrics, such as thickness, mass per square meter, and actual warp and weft density, were measured according to the standards. To determine the distance more accurately between the fabric and the 3D printer nozzle during the printing process, the thickness of the fabric was measured in two ways using a fabric thickness tester. Thickness 1 was measured according to the EN ISO 9863-1 standard using the standard circular pressure foot, while Thickness 2 was measured when the fabric was placed in the mounting frame prepared for 3D printing. The measured values of the fabric parameters are shown in Table 2.

The 3D models for the adhesion tests and microscopic observation were modeled in Blender 2.92 (Blender Foundation, Amsterdam, The Netherlands). The dimensions of the 3D model for T-peel adhesion were in accordance with standard requirements, 25 mm × 160 mm × 0.4 mm, and for morphology observation the dimensions were 15 mm × 15 mm × 0.2 mm. Models were exported as stl files to the slicing software Voxelizer (ZMorph, Wrocław, Poland), where parameters were set for 3D printing.

The G-codes were manually adjusted in a Notepad++ text and source code editor to obtain the exact z-distance and to avoid crashes of the printing nozzle into the mounting frame where the fabrics were placed.

**Figure 1:**
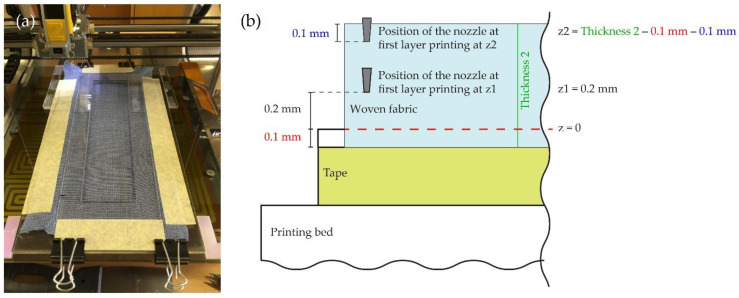
(**a**) Positioning of the mounting frame and installation on the printing bed; (**b**) Scheme of the z-distance calculation.

Since previous research has shown that proper mounting of the fabrics to the printer affects both the accuracy of the print and the time required to prepare the fabrics for 3D printing, we made custom mounting frames in which the fabrics were attached to the printing bed. The mounting frames and spacers were designed in Adobe Illustrator and cut from Plexiglas using a HyperCUT 6090 pro laser cutter (CNC stroji, Gorišnica, Slovenia). We placed and precisely positioned spacers on the printing bed, between which we inserted the mounting frame with a sample of fabric and fastened it with clamps (Figure 1a). In this way, we avoided the influence of differently positioned samples on the printing process.

The fabric samples were 3D printed using a 1.75 mm PLA filament (AzureFilm, Sežana, Slovenia) and a ZMorph 2.0 SX 3D FDM printer (ZMorph, Wrocław, Poland). The samples were printed in two layers for adhesion tests and one layer for morphology observation.

As mentioned in the Introduction and investigated in our preliminary tests, the distance between a printing nozzle and the fabric plays a very important role in the adhesion of 3D printed fabrics. We defined the exact zero position z = 0 with the measurement gap filler of 0.1 mm (Insize feeler gage with the range 0.02−1.0 mm, Insize CO., LTD, Suzhou New District, Suzhou, China). From the zero position, we determined the height of the print nozzle in two ways: the constant z-distance (z1) measured from z = 0 regardless of the thickness of the fabric, and the constant z-distance offset (z2) with respect to the fabric’s thickness (Figure 1b).

To observe the penetration of the polymer into the fabric substrate, we determined and printed at three different z-distances, namely 0.25 mm, 0.35 mm, and 0.45 mm.

Since the results of our preliminary tests confirmed the finding of the Kozior et al. study [17] that adhesion was the highest when the first layer was printed at a 90° angle to the longer size of the sample (the warp direction), and most fabrics tear too quickly in T-peel tests, we specified an infill angle of 45 degrees for printing the first layer. The printing parameters are listed in Table 3.

Figure 1a shows the positioning of the custom mounting frame and spacers with the inserted fabric attached to the 3D printer and (b) the graphical representation of the z-distance calculation.

### 2.2. Methods

T-peel adhesion tests were performed using an Instron 5567 dynamometer (Instron, Norwood, MA, USA). The tests were performed according to the standard DIN 53530 with a separation rate of 100 mm/min. According to the standard, three samples were measured in the warp direction.

The morphological properties of the fabrics and 3D printed objects on textile substrates were observed using a LEICA S9i optical microscope with Leica Application Suite 4.12.0 (Leica Microsystems GmbH, Wetzlar, Germany) and a JSM-6060LV scanning electron microscope (Jeol Ltd., Tokyo, Japan) (SEM). The samples for imaging in the SEM were coated with a thin gold film as a conductive coating using a JEOL JFC-1300 Coater (Jeol Ltd., Tokyo, Japan).

Designs of experiments (DOEs) for simple and double fabrics with predefined distinct factors in Statgraphics Centurion XV software (Statgraphics Technologies, Inc., The Plains, VA, USA) were created. For the simple and double woven fabrics, we specified two factors (weave structure and preset weft density on the loom) that affect the result—in our case, the adhesion force (Table 4). For each combination, we performed three repetitions.

However, we were interested in the influence of weave structure and density at different z-distances: at a constant z-distance (z1), when the nozzle enters the fabric differently during printing depending on the fabric’s thickness, and at a constant z-distance offset (z2) with respect to the fabric.

Four experiments of 3D printing on textile substrates were performed on fabrics with:

1. Simple weaves at a constant z-distance (z1);
2. Simple weaves at a constant z-distance offset (z2);
3. Double weaves at a constant z-distance (z1); and
4. Double weaves at a constant z-distance offset (z2).

For each experiment, 18 runs were performed. For the statistical analyses of the results, the multifactor ANOVA at a significance level of 0.05 was used. Two independent variables—factors with two and three levels—were selected for each experiment (Table 4).

## 3. Results and Discussion

In this section, we present and discuss the results of the adhesion force measurements and the results of the ANOVA statistical analysis. The morphological characteristics of the surface and cross-section of 3D printed fabrics with optical and scanning electron microscopy images, which directly affect the adhesion properties, are also presented below.

### 3.1. Adhesion Force Measurements

**Figure A1:**
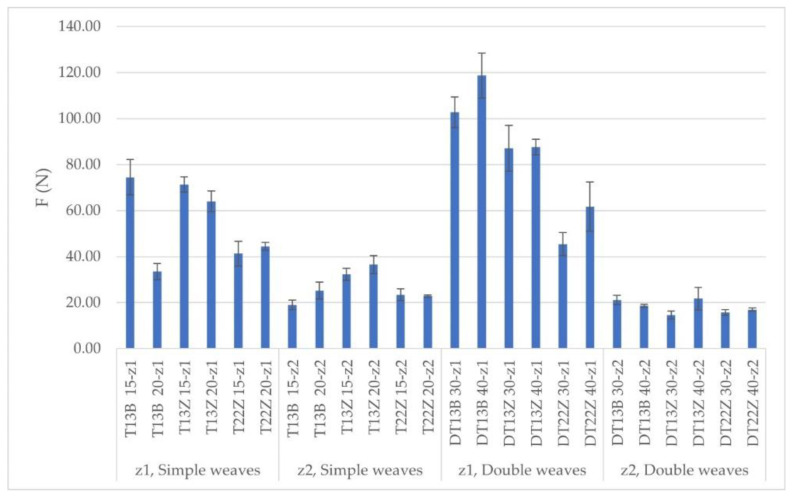
Average maximum adhesion force (F) and standard deviation for all samples of fabrics woven with simple and double weaves at different weave structures, weft densities, and z-distances.

Table 5 lists the average maximum adhesion forces for all 3D printed fabrics investigated, which are also presented in Figure A1. The results for simple fabrics at z1 (T13B, T13Z) were already presented in our preliminary study [23] where the value of z1 was calculated differently.

The highest adhesion force (118.7 N) was obtained for sample DT13B 40-z1 (double-weave fabric, broken twill, preset weft density 40 wefts/cm) printed at a constant z-distance (z1), and the lowest adhesion force (14.6 N) was achieved for sample DT13Z 30-z2 (double-weave fabric, twill 1/3, preset weft density 30 wefts/cm) printed at a constant z-distance offset (z2).

Some samples (T13B 15-z1, T13Z 15-z1, DT13B 30-z1, and DT13B 40-z1) achieved very high adhesion; therefore, they ruptured before the T-peel tests were completed.

Regarding the fabric construction (simple and double) and z-distance, the highest adhesion forces were generally obtained for double-weave fabrics (DT13B and DT13Z) printed with a constant z-distance (z1). The lowest values were also obtained for double-weave fabrics (DT13Z, DT13Z, and DT22Z) printed with a constant z-distance offset (z2). When comparing fabrics with simple and double weaves at a constant z-distance (z1), 3D printed fabrics with double weaves (DT13Z, DT13Z, and DT22Z) generally achieved higher adhesion forces; on the other hand, at a constant z-distance offset (z2), higher adhesion forces were measured for 3D printed fabrics with simple weaves (T13B, T13Z, and T22Z).

For fabrics with simple weaves and a constant z-distance (z1), the results show that fabrics with broken twill 1/3 and twill 1/3 Z achieve a higher adhesion force at a lower density (T13B 15 and T13Z 15). For samples woven in twill 2/2 Z with a higher density (T22Z 20), a slightly higher adhesion force was noticed. The highest adhesion force was achieved with the broken twill 1/3 weave with a preset weft density of 15 threads/cm (T13B 15). At a weft density of 20 threads/cm, the highest adhesion was achieved for the twill weave 2/2 Z (T22Z 20).

For fabrics with simple weaves and a constant z-distance offset (z2), the results show that 3D printed fabrics woven in broken twill 1/3 and twill 1/3 Z have a higher adhesion force at a higher preset weft density (T13B 20 and T13Z 20), and the opposite for samples woven in twill 2/2 Z, where slightly lower adhesion was noticed at a higher density (T22Z 20). The highest adhesion was achieved for the sample woven in twill 1/3 Z with a preset weft density of 20 threads/cm (T13Z 20), and the lowest adhesion was achieved for sample T13B 15, which was woven in broken twill 1/3 with a preset weft density of 15 threads/cm.

For fabrics with double weaves and a constant z-distance (z1), the results show that fabrics with all weave structures and a higher preset weft density result in a higher adhesion force. The adhesion force is the highest for sample DT13B 40 and the lowest for sample DT22Z 30. For the same fabrics and a constant z-distance offset (z2), fabrics woven in double twill 1/3 Z and double twill 2/2 Z with a higher density (DT13B 40 and DT13Z 40) also achieved a higher adhesion force. The fabric woven in double broken twill 1/3 had a lower adhesion force at a higher density (DT13B 40). The highest adhesion was achieved with the fabric woven in double weave with twill 2/2 and a preset weft density of 40 threads/cm (DT22Z 40). The lowest adhesion force was achieved with the fabric woven in double weave with twill 1/3 Z and a preset weft density of 30 threads/cm (DT13Z 30).

### 3.2. Statistical Analysis

#### 3.2.1. Influence on the Adhesion of 3D Printed Simple Fabrics at a Constant z-Distance (z1)

The ANOVA table (Table 6) shows that both main factors (weave and density) have a statistically significant influence on the adhesion of 3D printed simple fabrics at a constant z-distance (z1). According to the F-ratio, the influence of density is about 10% higher than the influence of weave.

**Figure 2:**
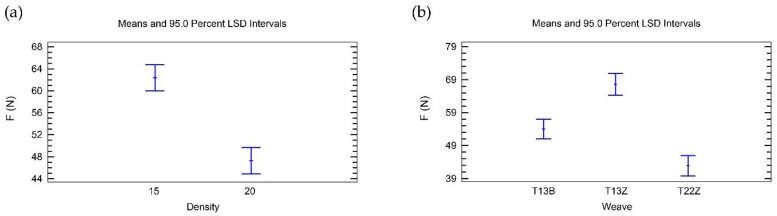
Influence of the main factors on the adhesion force for simple fabrics for z1: (**a**) density; (**b**) weave.

Figure 2a shows that the preset weft density of 15 wefts/cm has a greater influence on the adhesion force of 3D printed simple fabrics for z1 than the preset weft density of 20 threads/cm. Figure 2b shows that the twill weave 1/3 Z (T13Z) has the greatest influence on the adhesion force, the broken twill weave (T13B) has a smaller influence on the adhesion force, and the twill weave 2/2 Z (T22Z) has the smallest influence on the adhesion force.

The interaction between weave and density (Table 6) is also statistically significant, but it is the least important, as indicated by the lowest F-ratio. This was expected, since both factors are statistically significant and influence the adhesion of 3D printed simple fabrics at a constant z-distance (z1).

**Figure 3:**
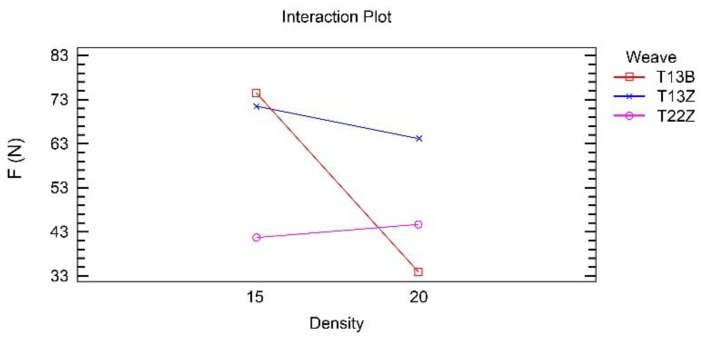
Interaction between density and weave in the analysis of the adhesion force for simple fabrics for z1.

Figure 3 shows that weave and density interact strongly; however, the T13B weave performs differently in combination with density than the other two weaves.

#### 3.2.2. Influence on the Adhesion of 3D Printed Simple Fabrics at a Constant z-Distance Offset (z2)

Table 7 shows that both factors (weave and density) have a statistically significant influence on the adhesion of 3D printed simple fabrics at a constant z-distance offset (z2). The influence of weave is approximately six times greater than the influence of density.

**Figure 4:**
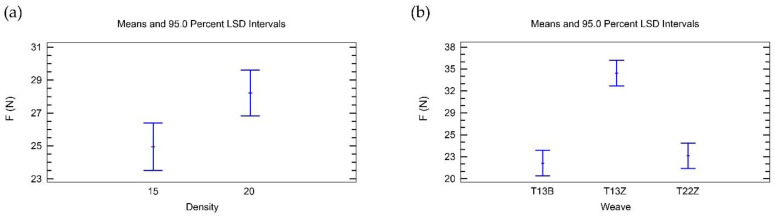
Influence of the main factors on the adhesion force for simple fabrics for z2: (**a**) density; (**b**) weave.

In contrast to the results for 3D printed simple fabrics at a constant z-distance (z1), Figure 4a shows that the weft density of 15 threads/cm has a smaller influence on the adhesion force of printed simple fabrics for a constant z-distance offset (z2) than the weft density of 20 threads/cm.

Figure 4b shows that the twill weave 1/3 Z (T13Z) has the highest influence on the adhesion force (the same as at z1). Broken twill (T13B) and twill 2/2 Z (T22Z) both have very similar influences on the adhesion force, but in this case the influence of broken twill (T13B) is the smallest.

The interaction between weave and density (Table 7) is not statistically significant, as the *p*-value is greater than 0.05.

#### 3.2.3. Influence on the Adhesion of 3D Printed Double Fabrics at a Constant z-Distance (z1)

The analysis of the adhesion of 3D printed double fabrics at a constant z-distance (z1) shows that the main factors, weave and density, have a statistically significant influence (Table 8). The influence of weave is approximately nine times greater than the influence of density.

**Figure 5:**
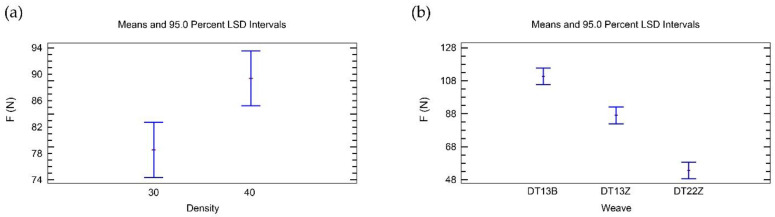
Influence of the main factors on the adhesion force for double fabrics for z1: (**a**) density; (**b**) weave.

Figure 5a shows that the weft density of 40 threads/cm has a higher influence on the adhesion force of printed double fabrics for a constant z-distance (z1) than the weft density of 30 threads/cm.

Figure 5b shows that double-weave broken twill 1/3 (DT13B) has the highest influence on the adhesion force, twill 1/3Z (DT13Z) has a smaller influence on the adhesion force, and twill 2/2 Z (DT22Z) has the smallest influence on the adhesion force.

The interaction between weave and density (Table 8) is not statistically significant.

#### 3.2.4. Influence on the Adhesion of 3D Printed Double Fabrics at a Constant z-Distance Offset (z2)

The statistical analysis of the adhesion of 3D printed double fabrics at a constant z-distance offset (z2) (Table 9) shows that there is no statistically significant main factor. Only the interaction between weave and density is statistically significant.

**Figure 6:**
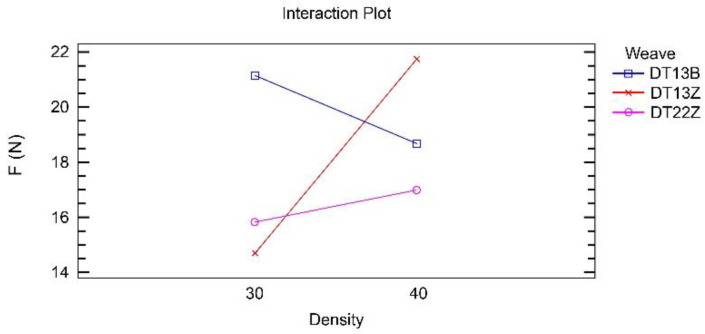
Interaction between density and weave in the analysis of the adhesion force for double fabrics for z2.

Figure 6 shows that weave and density interact strongly. The influence of this interaction is approximately twice as strong as the influence of the two main factors, weave and density, on the adhesion force.

The double twill weave 1/3 (DT13Z) has a different effect in combination with density compared with the other two weaves, as in the case of the 3D printed simple fabrics for a constant z-distance (z1).

### 3.3. Morphology of 3D Printed Fabrics

The morphological properties of fabrics and 3D printed objects on textile substrates were observed at different magnifications. We studied the morphology of fabrics 3D printed with one layer of PLA polymer at three different constant z-distances. Images were obtained using two microscopic methods: an optical microscope (the surface morphology observation) and a scanning electron microscope (the cross-sections).

**Figure A2:**
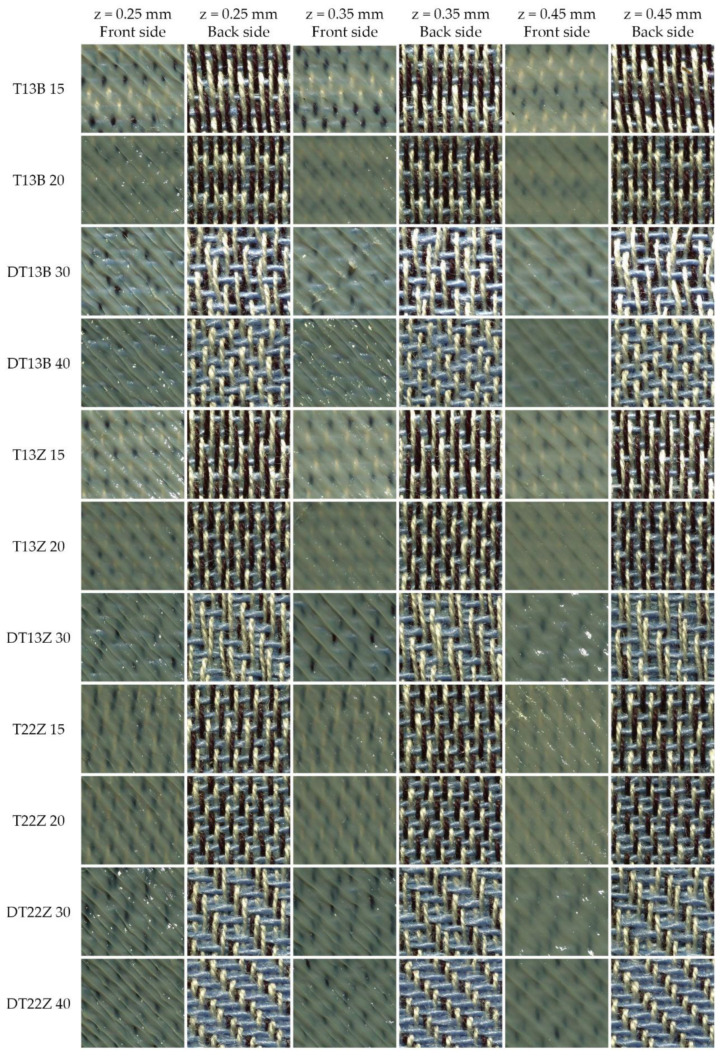
Front and back sides of the three-dimensional printed samples at different constant z-distances (mag. 20×).

Table 10 and Figure A2 show the surfaces of the fabrics and the printed fabrics at three different constant z-distances (z = 0.25 mm, z = 0.35 mm, and z = 0.45 mm). Images of the fabrics were taken with an optical microscope at 20× magnification.

Visual comparison of images of 3D printed fabric surfaces obtained with an optical microscope shows that the printed surface becomes smoother as the z-distance is increased for all fabric samples, regardless of the weave structure and the weft density, suggesting that the polymer’s penetration into the substrate is weaker when the print head is positioned higher (Table 10). Therefore, adhesion is expected to be lower as the polymer remains on the surface of the fabric. Moreover, the weave structure and thus the roughness of the fabric have an influence on the uniformity and smoothness of the printed surface due to the different thread floatations, thread arrangements, and numbers of interlacing points.

**Figure 7:**
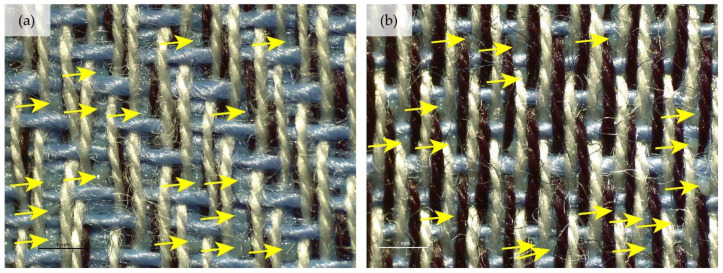
Images of the backs of printed fabric samples acquired with an optical microscope (mag. 20×) with marked deposits of molten polymer: (**a**) DT13Z 30, z = 0.25 mm; (**b**) T13Z 15, z = 0.25 mm.

When we look at the backs of the 3D printed fabric samples for fabrics with a lower weft density and the same weave pattern, we see more places where the polymer penetrates through the substrate. These are more numerous in the samples when the z-distance is smaller. Figure 7 shows back sides of printed fabric samples (sample DT13Z 30 and sample T13Z 15). Both samples were printed with the z-distance z = 0.25 mm. Numerous deposits of molten polymer, which have penetrated through the pores of the fabric, can be seen on both samples.

The following figures show the morphological properties of 3D printed fabrics at different magnifications based on images taken with a scanning electron microscope.

**Figure 8:**
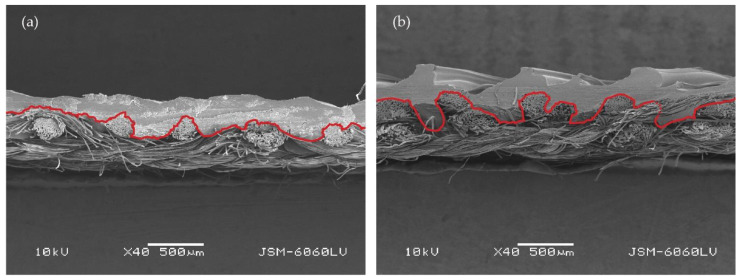
Cross-section (cut in the warp direction) of 3D printed fabrics at z = 0.25 mm (mag. 40×): (**a**) simple fabric T13Z 15; (**b**) double fabric DT13Z 30.

Figure 8 shows cross-sections (cut in the warp direction) of (a) the printed simple fabric T13Z 15 and (b) the printed double fabric DT13Z 30. The fabrics were printed with a z-distance of 0.25 mm. In the case of the double fabric, the density of the weft threads is higher, while the threads are arranged in two levels, the lower and upper thread layers, resulting in a grouping of threads depending on the weave; thus, the fabric structure is less compact. This is indicated by the red line in Figure 8a,b. The red line was drawn afterwards in the program Adobe Illustrator (Adobe Systems, San Jose, CA, USA) and only roughly indicates the boundary between the fabric surface and the polymer. In this way, it is easier to estimate the specific area of the two fabrics and to compare the red demarcation lines of simple and double fabrics. The longer the absolute length of the red line, the greater the specific surface area of the fabric, i.e., the greater the surface area to which the polymer adheres, resulting in greater adhesion. The length of the line representing the surface of the double fabric DT13Z 30 is approximately 20% longer than the length of the line representing the surface of the simple fabric T13Z 15 and this confirms the above statement.

**Figure 9:**
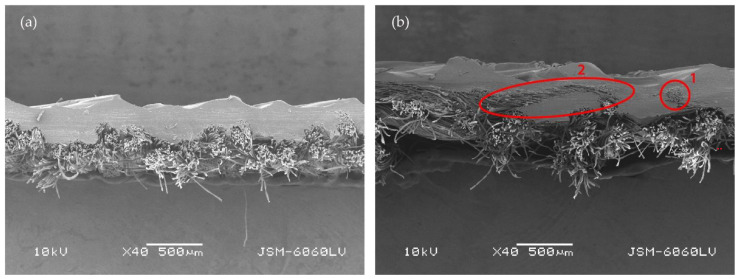
Cross-section (cut in the weft direction) of 3D printed fabrics at z = 0.25 mm (mag. 40×): (**a**) simple fabric T13Z 15; (**b**) double fabric DT13Z 30.

Figure 9 shows the cross-section (cut in the weft direction) of the 3D printed fabrics with a z-distance of 0.25 mm (magnification, 40×): (a) the simple fabric T13Z 15; and (b) the double fabric DT13Z 30. In the case of the DT13Z 30 sample, the polymer completely encloses the upper warp (label 1 in Figure 9b) and weft (label 2 in Figure 9b), while in the case of the simple fabric, the cross-section shows no threads completely enclosed by the polymer (Figure 8a).

**Figure 10:**
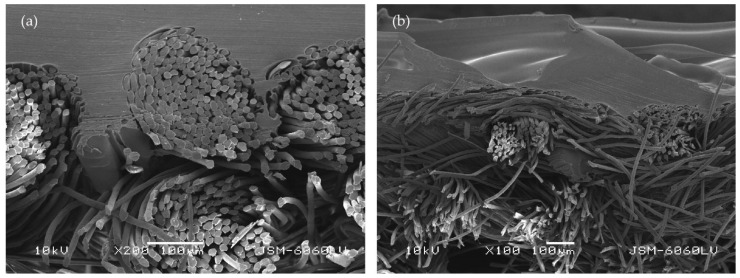
Cross-section of the 3D printed fabric DT13B 40 at z = 0.25 mm: (**a**) cut in the warp direction (mag. 200×); (**b**) cut in the weft direction (mag. 100×).

Figure 10 shows a cross-section of the double fabric DT13B 40 (a) along the warp at a magnification of 200× and (b) along the weft at a magnification of 100×. Image (a) shows how the polymer surrounds the weft yarn (PES) and how it penetrates into the yarn and surrounds the individual fibers. Image (b) shows how the polymer surrounds the warp yarn (cotton).

## 4. Conclusions

The main objective of our research was to compare the influence of a simple and a double fabric construction on the adhesion of a 3D printed polymer to the substrate using FDM technology. We focused on the influence of the weave pattern and the weft density. We studied the influence of three derivatives of four-end twill and four different weft densities (two for the simple fabric and two for the double fabric). The study was performed on samples printed with two different z-distance settings. One part of each sample was printed with a constant z-distance, and the other part was printed with a constant z-distance offset from the fabric surface.

In explaining the results, we also relied on the second part of the study, where we examined the surface and cross-section of the printed samples using an optical microscope and a scanning electron microscope.

At the constant z-distance (z1), all samples had higher adhesion strength than at the constant z-distance offset (z2). This result was expected, as, with z1, the printer nozzle penetrates deeper into the fabric; depending on the fabric’s thickness, the polymer penetrates more deeply into the fabric and wraps around the yarn and the individual fibers. However, when printing with a constant z-distance (z1), simple fabrics have poorer adhesion than thicker double fabrics. The printer nozzle penetrates more into the double fabric, and the polymer penetrates through the pores of the upper layer into the lower layer, where it adheres to the yarn or fibers. In addition, double fabrics have a higher thread density, but the threads are arranged in two layers and grouped according to the weave. As a result, the structure of the fabric is less compact, the specific surface area is larger, and thus the adhesion is better.

Comparing the adhesion of simple and double 3D printed fabrics with a constant z-distance offset (z2), where the thread arrangement in the fabric has a greater influence, the adhesion of simple fabrics is generally higher than that of double fabrics. At z2, the printing nozzle goes only 0.1 mm into the fabric and, in the case of double weaves, does not reach the bottom layer at all. Since both the warp and weft threads are divided into two layers, the upper layer of double fabrics has a smaller number of warp threads, so the surface to which polymer can adhere is smaller than at simple fabrics, resulting in a smaller adhesion force.

In summary, 3D printed double fabrics, which allow for the use of different raw materials in the upper and lower layers of the fabric, exhibit a great deal of potential. With the right fabric construction parameters, as well as controlled printing conditions, better adhesion of the 3D printed polymer to the textile substrate can be achieved.

## Figures and Tables

**Table 1:** Woven fabric samples (mag. 20×) with their designations (the sample designation includes the name of the weave structure and the preset number of threads per centimeter).

Weave/On-Loom Setting	Broken Twill 1/3	Twill 1/3 Z	Twill 2/2 Z
Simple weaves	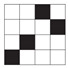 T13B	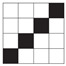 T13Z	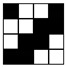 T22Z
Density 15 threads/cm	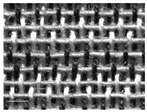 T13B 15	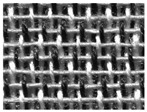 T13Z 15	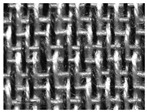 T22Z 15
Density 20 threads/cm	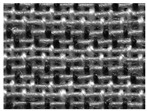 T13B 20	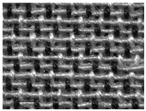 T13Z 20	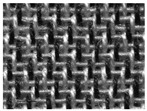 T22Z 20
Double weaves	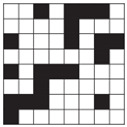 DT13B	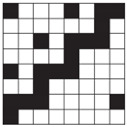 DT13Z	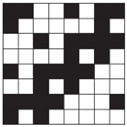 DT22Z
Density 30 threads/cm	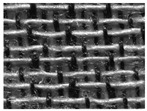 DT13B 30	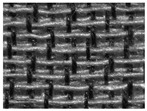 DT13Z 30	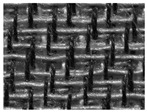 DT22Z 30
Density 40 threads/cm	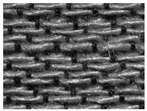 DT13B 40	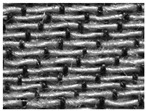 DT13Z 40	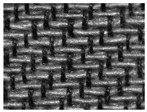 DT22Z 40

**Table 2:** Measured values of physical properties of woven fabrics used as substrates for 3D printing (Thickness 1 and Thickness 2 (mm), thickness difference (mm, %), warp (threads/cm) and weft density (threads/cm), and mass per unit area (g/m^2^)).

Sample	Thickness 1	Thickness 2	Thickness Difference		Mass	Warp Density	Weft Density
	(mm)	(mm)	(mm)	(%)	(g/m^2^)	(Threads/cm)	(Threads/cm)
T13B 15	0.508	0.430	−0.078	−15.35	114.0	40.7	15.0
T13B 20	0.473	0.408	−0.065	−13.74	139.8	40.6	21.7
T13Z 15	0.489	0.423	−0.066	−13.50	112.8	40.4	14.6
T13Z 20	0.487	0.430	−0.057	−11.70	140.4	40.6	22.3
T22Z 15	0.527	0.447	−0.080	−15.18	112.6	40.8	14.4
T22Z 20	0.490	0.445	−0.045	−9.18	139.4	40.5	22.1
DT13B 30	0.743	0.615	−0.128	−17.23	168.6	41.7	31.2
DT13B 40	0.756	0.690	−0.066	−8.73	230.2	41.9	44.1
DT13Z 30	0.747	0.628	−0.119	−15.93	168.4	41.3	31.5
DT13Z 40	0.752	0.680	−0.072	−9.57	228.8	41.9	46.1
DT22Z 30	0.704	0.598	−0.106	−15.06	164.6	41.2	31.2
DT22Z 40	0.688	0.610	−0.078	−11.34	231.0	41.7	46.5

**Table 3:** Three-dimensional printing parameters.

Parameter	Value
Nozzle size	0.4 mm
Layer height	0.2 mm
Infill print speed	40 mm/s
Infill angle	45°
Outline print speed	30 mm/s
Outline count	2
Extruder temperature	210 °C
Bed temperature	60 °C

**Table 4:** Factors of statistical designs of experiments for simple and double weaves.

Factors	Levels	Units	Simple Weaves	Double Weaves
Weave	3	Weave structure	T13B, T13Z, T22Z	DT13B, DT13Z, DT22Z
Density	2	Weft density (threads/cm)	15, 20	30, 40

**Table 5:** Average maximum adhesion force (F), standard deviation (SD), and coefficient of variation (CV) for all samples of fabrics woven with simple and double weaves at different weave structures, weft densities, and z-distances.

	Simple Weaves				Double Weaves			
Z	Sample	F (N)	SD (N)	CV (%)	Sample	F (N)	SD (N)	CV (%)
z1	T13B 15-z1	74.5	7.7	10	DT13B 30-z1	102.8	6.7	6.5
	T13B 20-z1	33.4	3.6	11	DT13B 40-z1	118.7	9.7	8.2
	T13Z 15-z1	71.4	3.4	4.7	DT13Z 30-z1	87	10	11
	T13Z 20-z1	64.0	4.5	7.0	DT13Z 40-z1	87.6	3.5	4.0
	T22Z 15-z1	41.3	5.3	13	DT22Z 30-z1	45.4	4.9	11
	T22Z 20-z1	44.5	1.8	4.0	DT22Z 40-z1	62	11	17
z2	T13B 15-z2	19.0	2.0	10	DT13B 30-z2	21.1	2.0	9.5
	T13B 20-z2	25.2	3.7	15	DT13B 40-z2	18.64	0.70	3.7
	T13Z 15-z2	32.3	2.6	8.1	DT13Z 30-z2	14.6	1.7	11
	T13Z 20-z2	36.6	3.9	11	DT13Z 40-z2	21.7	4.9	23
	T22Z 15-z2	23.4	2.6	11	DT22Z 30-z2	15.8	1.3	8.2
	T22Z 20-z2	22.85	0.48	2.1	DT22Z 40-z2	16.93	0.64	3.8

**Table 6:** Impact of weave and density on the adhesion force for simple fabrics for z1.

Source	Sum of Squares	Df	Mean Square	F-Ratio	*p*-Value
MAIN EFFECTS					
A: Weave	1859.67	2	929.835	41.45	0.0000
B: Density	1023.21	1	1023.21	45.61	0.0000
INTERACTIONS					
AB	1601.03	2	800.513	35.68	0.0000
RESIDUAL	269.214	12	22.4345		
TOTAL (CORRECTED)	4753.12	17			

Df—Degrees of freedom.

**Table 7:** Impact of weave and density on the adhesion force for simple fabrics for z2.

Source	Sum of Squares	Df	Mean Square	F-Ratio	*p*-Value
MAIN EFFECTS					
A: Weave	562.655	2	281.328	36.38	0.0000
B: Density	48.8171	1	48.8171	6.31	0.0273
INTERACTIONS					
AB	36.4052	2	18.2026	2.35	0.1373
RESIDUAL	92.8029	12	7.73358		
TOTAL (CORRECTED)	740.681	17			

**Table 8:** Impact of weave and density on the adhesion force for double fabrics for z1.

Source	Sum of Squares	Df	Mean Square	F-Ratio	*p*-Value
MAIN EFFECTS					
A: Weave	9904.5	2	4952.25	76.46	0.0000
B: Density	533.915	1	533.915	8.24	0.0141
INTERACTIONS					
AB	244.868	2	122.434	1.89	0.1934
RESIDUAL	777.246	12	64.7705		
TOTAL (CORRECTED)	11,460.5	17			

**Table 9:** Impact of weave and density on the adhesion force for double fabrics for z2.

Source	Sum of Squares	Df	Mean Square	F-Ratio	*p*-Value
MAIN EFFECTS					
A: Weave	37.7537	2	18.8769	3.36	0.0696
B: Density	16.7582	1	16.7582	2.98	0.1100
INTERACTIONS					
AB	70.5002	2	35.2501	6.27	0.0137
RESIDUAL	67.5145	12	5.62621		
TOTAL (CORRECTED)	192.527	17			

**Table 10:** Images of fabric samples (mag. 20×) printed at different z-distances (0.25 mm, 0.35 mm, and 0.45 mm).

Sample	z-Distance		
	z = 0.25 mm	z = 0.35 mm	z = 0.45 mm
T13B 15	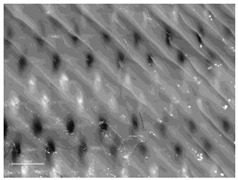	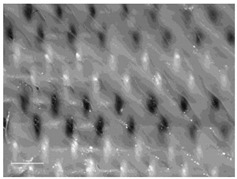	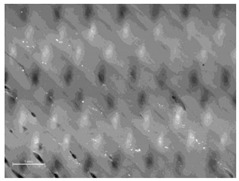
DT 13Z 40	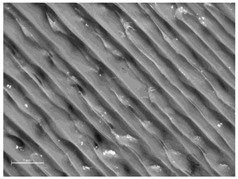	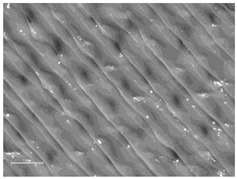	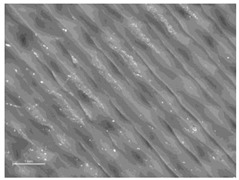

## Data Availability

The data presented in this study are available on request from the corresponding author.

## References

[1] Dip T.M., Emu A.S., Nafiz M.N.H., Kundu P., Rakhi H.R., Sayam A., Akhtarujjman M., Shoaib M., Ahmed M.S., Ushno S.T. (2021). 3D printing technology for textiles and fashion. Text. Prog..

[2] Gibson I., Rosen D., Stucker B. (2015). Introduction and Basic Principles. Additive Manufacturing Technologies: 3D Printing, Rapid Prototyping, and Direct Digital Manufacturing.

[3] Dave H.K., Patel S.T., Dave H.K., Davim J.P. (2021). Introduction to Fused Deposition Modeling Based 3D Printing Process. Fused Deposition Modeling Based 3D Printing.

[4] Melnikova R., Ehrmann A., Finsterbusch K., Broitman E. (2014). 3D printing of textile-based structures by Fused Deposition Modelling (FDM) with different polymer materials. IOP Conference Series-Materials Science and Engineering, Proceedings of the 2014 Global Conference on Polymer and Composite Materials, Ningbo, China, 27–29 May 2014.

[5] Sitotaw D.B., Ahrendt D., Kyosev Y., Kabish A.K. (2020). Additive Manufacturing and Textiles—State-of-the-Art. Appl. Sci..

[6] Tümer E.H., Erbil H.Y. (2021). Extrusion-Based 3D Printing Applications of PLA Composites: A Review. Coatings.

[7] Muck D., Križanovskij I. (2015). 3D-Tisk.

[8] Sin L.T., Tueen B.S., Sin L.T., Tueen B.S. (2019). 10-Injection Molding and Three-Dimensional Printing of Poly(Lactic Acid). Polylactic Acid (Second Edition).

[9] Korger M., Bergschneider J., Lutz M., Mahltig B., Finsterbusch K., Rabe M., Aumann S., Ehrmann A., Weber M.O. (2016). Possible Applications of 3D Printing Technology on Textile Substrates. IOP Conference Series-Materials Science and Engineering, Proceedings of the 48th Conference of the International Federation of Knitting Technologists, Moenchengladbach, Germany, 8–11 June 2016.

[10] Ahrendt D., Karam A.R. (2020). Development of a computer-aided engineering-supported process for the manufacturing of customized orthopaedic devices by three-dimensional printing onto textile surfaces. J. Eng. Fiber. Fabr..

[11] Awaja F., Gilbert M., Kelly G., Fox B., Pigram P. (2009). Adhesion of polymers. Prog. Polym. Sci..

[12] Grimmelsmann N., Kreuziger M., Korger M., Meissner H., Ehrmann A. (2018). Adhesion of 3D printed material on textile substrates. Rapid Prototyp. J..

[13] Sabantina L., Kinzel F., Ehrmann A., Finsterbusch K. (2015). Combining 3D printed forms with textile structures mechanical and geometrical properties of multi-material systems. IOP Conference Series-Materials Science and Engineering, Proceedings of the 2015 Global Conference on Polymer and Composite Materials, Beijing, China, 16–18 May 2015.

[14] Kočevar T.N., Drusany M. Designing a pattern with 3D printing on textiles. Proceedings of the Book of proceedings XVth International İzmir Textile and Apparel Symposium.

[15] Korger M., Glogowsky A., Sanduloff S., Steinem C., Huysman S., Horn B., Ernst M., Rabe M. (2020). Testing thermoplastic elastomers selected as flexible three-dimensional printing materials for functional garment and technical textile applications. J. Eng. Fiber. Fabr..

[16] Spahiu T., Al-Arabiyat M., Martens Y., Ehrmann A., Piperi E., Shehi E. (2018). Adhesion of 3D printing polymers on textile fabrics for garment production. IOP Conference Series: Materials Science and Engineering, Proceedings of the Aegean International Textile and Advanced Engineering Conference, Lesvos, Greece, 5–7 Septembre 2018.

[17] Kozior T., Doepke C., Grimmelsmann N., Junger I.J., Ehrmann A. (2018). Influence of fabric pretreatment on adhesion of three-dimensional printed material on textile substrates. Adv. Mech. Eng..

[18] Spahiu T., Grimmelsmann N., Ehrmann A., Piperi E., Shehi E. Effect of 3D printing on textile fabric. Proceedings of the 1st International Conference “Engineering and Entrepreneurship”.

[19] Malengier B., Hertleer C., Cardon L., Van Langenhove L. (2018). 3D Printing on Textiles: Testing of Adhesion. J. Fash. Technol. Text. Eng..

[20] Unger L., Scheideler M., Meyer P., Harland J., Goerzen A., Wortmann M., Dreyer A., Ehrmann A. (2018). Increasing Adhesion of 3D Printing on Textile Fabrics by Polymer Coating. Tekstilec.

[21] Narula A., Pastore C.M., Schmelzeisen D., El Basri S., Schenk J., Shajoo S. (2018). Effect of knit and print parameters on peel strength of hybrid 3-D printed textiles. J. Text. Fibrous Mater..

[22] Görmer D., Störmer J., Ehrmann A. (2020). The influence of thermal after-treatment on the adhesion of 3D prints on textile fabrics. Commun. Dev. Assem. Text. Prod..

[23] Čuk M., Bizjak M., Muck D., Kočevar T.N. 3D printing and functionalization of textiles. Proceedings of the 10th International Symposium GRID.

